# Nanopore Sequencing of *Amoebophrya* Species Reveals Novel Collection of Bacteria Putatively Associated With *Karlodinium veneficum*

**DOI:** 10.1093/gbe/evaf022

**Published:** 2025-02-13

**Authors:** Daniela Tizabi, Russell T Hill, Tsvetan Bachvaroff

**Affiliations:** Institute of Marine and Environmental Technology, University of Maryland Center for Environmental Science, Baltimore, MD, USA; Institute of Marine and Environmental Technology, University of Maryland Center for Environmental Science, Baltimore, MD, USA; Institute of Marine and Environmental Technology, University of Maryland Center for Environmental Science, Baltimore, MD, USA

**Keywords:** dinoflagellate, MAG, marine alveolate, endoparasite, syndinean

## Abstract

The dinoflagellate parasite *Amoebophrya* sp. ex *Karlodinium veneficum* plays a major role in controlling populations of the toxic bloom-forming dinoflagellate *K. veneficum* and is one of the few cultured representatives of Marine Alveolate Group II. The obligate parasitic nature of this *Amoebophrya* spp. precludes isolation in culture, and therefore, genomic characterization of this parasite relies on metagenomic sequencing. Whole-genome sequencing of an *Amoebophrya* sp. ex *K. veneficum-*infected culture using Nanopore long reads revealed a diverse community of novel bacteria as well as several species previously reported to be associated with algae. In sum, 39 metagenome-assembled genomes were assembled, and less than half of these required binning of multiple contigs. Seven were abundant but of unknown genera, 13 were identifiable at the generic level by BLAST (8 of which were apparently complete single-contig genomes), and the remaining 19 comprised less abundant (individually accounting for <2% of the total bacterial reads in the culture) and often rarer and/or novel species. Attempts to culture strains identified through sequencing revealed that only two of these bacterial isolates were readily amenable to cultivation, stressing the importance of a dual culture- and sequencing-based approach for robust community analysis. Functional annotations of metagenome-assembled genomes are presented here to support the characterization of a microbial community associated with *K. veneficum* and/or *Amoebophrya* sp. ex *K. veneficum* cultured from the Chesapeake Bay and give preliminary insights into the nature of the associations these bacteria have with this parasite–host complex.

**Significance**

The obligate dinoflagellate parasite *Amoebophrya* sp. ex *Karlodinium veneficum*, one of the few cultured members of Marine Alveolate Group II, plays a major role in controlling populations of the toxic bloom-forming dinoflagellate *K. veneficum.* Little is known about the bacteria associated with *Amoebophrya* sp., and Nanopore whole-genome sequencing of the *Amoebophrya* sp. culture and host alone revealed a diverse bacterial community of 39 metagenome-assembled genomes (MAGs), several of which have no available reference genomic data and represent novel, uncultured bacteria. Genomic analysis of metabolic pathways of these bacterial strains represents a relatively complete microbial genomic survey of *Amoebophrya* sp. ex *K. veneficum* in culture.

## Introduction

*Amoebophrya* sp. ex *Karlodinium veneficum* (previously referred to as *Amoebophrya* sp. ex *K. micrum*) is an endoparasitic dinoflagellate known to infect the toxic bloom-forming dinoflagellate *K. veneficum*. *Karlodinium veneficum* is widespread throughout coastal marine environments including, but not limited to, the United States, Southwest Africa, Europe, Asia, and Western Australia (Copenhagen 1954; Bjørnland and Tangen 1979; Delgado et al. 1995; Gunderson et al. 2002; Zhang et al. 2008; Place et al. 2012; Adolf et al. 2015). Under conditions of excess nutrients and reduced dispersal, this toxic dinoflagellate is capable of forming harmful algal blooms (HABs) resulting in destructive fish kills (Hall et al. 2008; Place et al. 2012). These toxic effects are attributed to the production of lipophilic compounds with hemolytic, cytotoxic, and ichthyotoxic properties (known collectively as karlotoxins) (Sola et al. 1999; Deeds et al. 2002; Adolf et al. 2006; Bachvaroff et al. 2008; Zhang et al. 2008; Cai et al. 2016). In addition to their toxic activities, karlotoxins act as grazing deterrents, enabling *K. veneficum* to compete with other dinoflagellates as well as immobilize prey to facilitate their mixotrophic lifestyle (Li et al. 1996; Adolf et al. 2006, 2007; Bachvaroff et al. 2008; Hall et al. 2008).

*Amoebophrya* sp. ex *K. veneficum* is part of a diverse genus that, in culture, ranges from nonhost specific to extremely host specific (Coats et al. 1996; Coats and Park 2002; Kim 2006). This parasite is host specific and is only known to reproduce in culture on *K. veneficum*, and, through a predatory relationship, is able to control host populations and thus toxic bloom formation (Coats and Park 2002; Bachvaroff et al. 2009; Bachvaroff 2019). *Amoebophrya* is the sole genus described in a massive rDNA clade called the Marine Alveolate Group II or Marine Alveolate Lineages II (MALV-II) that is detected at high abundances and diversities in most marine environmental surveys (Gunderson et al. 2002; Moreira and López-García 2002; Skovgaard et al. 2005; Guillou et al. 2008; Kim et al. 2008). MALV-II ranks among the most abundant taxa both in pico-nanoplankton and in mesoplankton sampled in the Tara Oceans expedition (de Vargas et al. 2015) and could play a significant role in carbon cycling via host lysis and remineralization (Guillou et al. 2008, Hu et al. 2021, Anderson et al. 2024).

In sequencing the genome of *Amoebophrya* sp. ex *K. veneficum*, a nearly equal amount of data pertaining to bacterial species was also assembled, mostly as single-contig metagenome-assembled genomes (MAGs). As there are no other studies investigating microbial communities of either the parasitic or host dinoflagellate, this created an opportunity to describe the prokaryotic sequence data and thus the bacteria associated with cultured *K. veneficum* and *Amoebophrya*-infected *K. veneficum* derived from the Chesapeake. The approach used here employed bulk DNA isolation and Oxford Nanopore MinION long-read sequencing on late-stage *Amoebophrya*-infected *K. veneficum* cultures after host lysis. The *Amoebophrya* genome is ∼130 Mb (Bachvaroff 2019), the host genome size is between 5.5 Gb by UV spectrophotometry (Liu et al. 2021) and 21.9 Gb by flow cytometry (Figueroa et al. 2010), and the prokaryotic component of the community is unknown. Here, the prokaryotic, culture-based MAGs are described and validated, and their roles in the culture system are investigated.

## Results

### Creating a Reference Set of Bacterial Contigs MAGs

From sequencing *Amoebophrya*-infected *K. veneficum* cultures, we identified contigs up to 7 Mb in length containing 16S ribosomal RNA (rRNA) gene sequences of bacterial origin that were consistently generated using both Flye and Canu assemblers. Similarly, 16S rRNA gene-containing contigs were identified by sequencing uninfected *K. veneficum*; however, sequencing depth and effort were significantly reduced. The mixed host/parasite culture was sequenced six times, each time ∼1 month apart, and yielded 3.8 million bacterial reads (with an average error rate of 0.05) compared with the naïve host culture, which generated only 28,000 bacterial reads during a single sequencing run. Therefore, sequencing data from the mixed host/parasite culture were primarily used to assemble and assess these prokaryotic MAGs. The bacterial reads from the mixed host/parasite culture were assembled and binned based on high-identity 16S rRNA gene sequence and the MaxBin2 tool into a total of 39 MAGs with unique 16S rRNA genes. “MAG” will be used to describe any contig or contigs that bin together based on 16S gene rRNA gene sequence identity or the MaxBin2 tool. Of these 39 MAGs, 16 contained multiple contigs, and the remaining 23 were single-contig, apparently complete, bacterial genomes. The prokaryotic reference data presented here span 163 Mb and represent the longest nonoverlapping bacterial contigs from both mixed host/parasite and naïve host cultures, as well as between the Flye and Canu assemblies.

### Assessing Diversity and Composition of the Microbial Community

**Fig. 1.**
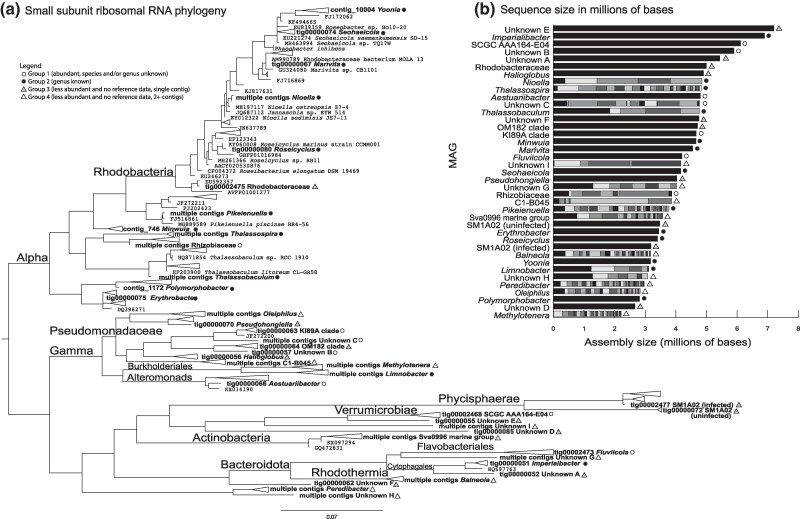
a) Maximum likelihood phylogenetic tree based on 16S rRNA gene sequences of cooccurring bacteria identified in *Amoebophrya*-infected *K. veneficum* and uninfected *K. veneficum* cultures. Bold taxon names are from this study, and remaining sequences were obtained from SILVA as “neighbor” sequences in the ACT pipeline. b) Length of bacterial contigs assembled in ascending order with differently shaded segments represents individual contigs within a bin. The taxon labels for b) are derived from NCBI BLAST descriptions in the case of >98% identity of 16S rRNA gene sequence or SILVA in the case of <98% identity. Nine 16S rRNA gene sequences could not be classified by SILVA and are labeled as “Unknown A to I.”

Phylogenetic analysis of 16S rRNA genes identified during metagenome assembly revealed a highly diverse bacterial community cooccurring with *K. veneficum* and/or the *Amoebophrya* parasite (Fig. 1). The 39 distinct 16S rRNA genes identified were comprised of mainly Pseudomonadota (13 Alphaproteobacteria and 9 Gammaproteobacteria), as well as Bacteroidota (3), Verrucomicrobiota (1), Planctomycetota (2), Actinobacteriota (1), Bdellovibrionota (1), and 9 16S rRNA gene sequences of unknown phyla based on the SILVA (http://www.arb-silva.de) Silva INcrimental Aligner/Alignment Classifcation and Tree (SINA/ACT) pipeline. This pipeline includes three prokaryotic taxonomy classifiers: the Ribosomal Database Project, European Molecular Biology Laboratory, and the European Bioinformatics Institute. Of the 39 total MAGs, 21 bacterial 16S rRNA gene sequences are identifiable by SILVA to the generic level (Fig. 1a). All MAGs assembled to at least 2 Mb in length (Fig. 1b). Several MAGs were identified as containing multiple 16S gene rRNA copies that slightly differed, in which case the copy with the highest BLAST identity was selected for identification. Specifically, although *Thalassospira* is displayed as one branch on the tree, four unique, slightly varying 16S rRNA gene sequences, were identified from this genus, suggesting multiple *Thalassospira* species present in the community or variation between 16S rRNA gene copies within the genome. Three MAGs (*Roseitalea stylonematis*, UBA7803 sp., and *Yoonia* sp. 018402175, as annotated by Genome Taxonomy Database [GTDB]) assembled solely from naïve host data due to high abundance in the uninfected *K. veneficum* culture, but these MAGs were able to recruit reads from the mixed culture using read mapping. Due to relatively higher depth of sequencing, the remaining 36 MAGs were drawn from the mixed host/parasite culture.

Additional taxonomic classification with the GTDB (Parks et al. 2022) revealed that the nine elusive bacterial sequences with poor assignment based on 16S rRNA gene sequence belong to Bacteroidota (3), Pseudomonadota (2, both Gammaproteobacteria), Verrucomicrobiota (2), Bdellovibrionota (1), and Chlamydiota (1) while confirming the higher level taxonomic assignments of the remaining MAGs as identified by 16S rRNA genes. Based on the GTDB database, only 14 of the 39 MAGs had average nucleotide identity (ANI) identities over 95%, of which eight were identified to named species, four to family, and two to order. These results are roughly mirrored by the more detailed protein-coding gene taxonomy assignment for MAGs without ANI identity. Overall, the GTDB protein assignment to described taxonomic groups identified 8 MAGs to species, 6 to genus, 17 to family, 7 to order, and 1 to a named class. However, for the informally described ranks in the GTDB taxonomy, typically with the uncultured bacterial affiliation (UBA) acronym, only one MAG was unidentified and could only be classified in this current release as belonging to the Alteromonadaceae family but is here operationally labeled as *Aestuariibacter* (based on SILVA ID using 16S rRNA gene sequence).

### Dividing the MAGs into Categories

The 39 MAGs are divided into a total of four categories. The first group includes seven most abundant MAGs not identified below the genus level based on 16S rRNA gene sequences and includes several apparently novel taxa (Table 1). The genome sizes of these Group 1 MAGs range from 3.9 to 6.1 Mb with sequencing depth from 9- to 350-fold. All had high estimated completeness, and all but one had low levels of contamination (one was above 7%). The second group contains 13 sequences from 2.8 to 6.9 Mb that could be identified to the generic level and had high-quality reference data for genome scale comparisons (Table 2; supplementary table S1, Supplementary Material online). The remaining 19 sequences that were not as abundant and lacked a good reference genome were then divided based on the extent of binning required for their assemblies into the third and fourth categories. The third category consists of 10 MAGs with a size between 2.6 and 7.2 Mb, all of which are composed of single contigs (Table 3). The fourth category includes nine low abundance MAGs (2.2 to 4.2 Mb) that require more extensive binning (each comprising 6 to 33 contigs) and are treated only in supplementary table S2, Supplementary Material online. Some of these 19 less abundant and incomplete MAGs may be derived from contamination introduced during sample processing, and their identity cannot be resolved with confidence based on their 16S rRNA gene sequence. Therefore, we refrain from speculating on the significance of their individual associations within the dinoflagellate cultures and limit their descriptions to Table 3 and supplementary tables. Operational taxon labels for all bacteria discussed are provided from NCBI BLAST descriptions in the case of >98% identity of 16S rRNA gene sequence and from SILVA in the case of <98% identity moving forward.

**Table 1:** Most abundant contigs identified as unknown or uncultured genera from *Amoebophrya*-infected and uninfected *K. veneficum* cultures

SILVA ID (GenBank accession no.)	Culture	Coverage^a^	ID (%)^b^	Contig length^c^	# Contigs	Completeness (%)	Contamination (5)	GC (%)	Coding Sequences	tRNA	rRNA	GTDB taxonomy
*Aestuariibacter* (CP170457)	Infected	350	95.7	4,727,878	1	99.1	1.3	45	4,334	48	12	(Pseudomonadota) Alteromonadaceae
SCGC AAA164-E0 (CP170459)	Infected	133	87.2	6,080,780	1	99	1	51	5,324	48	4	(Verrucomicrobiota) AAA164-E04 sp027619545
Unclassified (Unknown B) (CP170502)	Infected	87	89.1	5,853,790	1	100	0	57	5,323	40	3	(Pseudomonadota) HKST-UBA223
Rhodobacteraceae (CP170368)	Infected	111	95.6	4,945,740	1	99.6	1.9	68	4,898	50	3	(Pseudomonadota) ASV31 sp019061145
Unclassified (Unknown C, PRJNA1103200)^d^	Uninfected	9	90.3	4,774,814	18	100	0	68	5,007	49	3	(Pseudomonadota) UBA7803
KI89A clade (CP170489)	Uninfected	16	88.4	4,636,606	1	100	0	55	4,472	37	3	(Pseudomonadota) UBA4421
Rhizobiaceae (PRJNA1103200)^d^	Uninfected	10	97.3	3,865,668	9	100	7.7	64	4,134	42	3	(Pseudomonadota) *R. stylonematis*

“Culture” refers to the culture in which the bacterium is more abundant. All of these bacteria, except for the Rhizobiaceae and “Unknown C,” were identified in both infected and uninfected *K. veneficum* cultures. All remaining MAGs were assembled with Canu.

^a^Mean reads per base.

^b^Percent identity using BLASTn and the NCBI rRNA_typestrains/16S_ribosomal_RNA database.

^c^Single contig length minus any overlap.

^d^MAGs with the SILVA IDs “Rhizobiaceae” and “Unknown C” could only be assembled with Flye, as they did not have enough coverage for Canu assembly.

**Table 2:** MAGs identified from *Amoebophrya*-infected *K. veneficum* culture with a minimum of 98% 16S rRNA gene BLASTn identity

MAG BLAST ID (GenBank accession no.)	Coverage^b^	Flye ID (%)^c^	Canu ID (%)^c^	Flye length	Canu length	Flye # contigs	Canu # contigs	Typical genome size (Mb)^d^	GTDB taxonomy
*E. aurantius* strain C5* (CP180349)	354	98.9	98.9	3,328,698	**3,359,381**	1	1	∼3.4	(Pseudomonadota) *E. aurantius*
*Marivita* sp. CB1101* (CP180351)	80	99.6	99.6	4,656,275	**4,526,901**	2	1	3.6 to 4.9	(Pseudomonadota) *Marivita* sp030148515
*Imperialibacter* sp. Rs* (CP180355)	42	99.2	99.2	6,856,966	**6,856,909**	1	1	4.8 to 6.8	(Bacteroidota) *Imperialibacter*
*Limnobacter thiooxidans* CS-K2 (PRJNA1103200)	16	99.3	99.3	**3,118,024**	2,941,441	6	23	∼3.5	(Pseudomonadota) *Limnobacter* sp902506325
*Minwuia thermotolerans* SY3-13* (CP180352)	58	97.8	97.8	**4,635,690**	4,611,058	1	1	4.6 to 4.9	(Pseudomonadota) *Minwuia*
*Nioella ostreopsis* Z7-4 (JBLLNQ000000000)	59	99.8	99.8	4,923,006	**4,850,388**	10	5	∼4.6	(Pseudomonadota) *N. ostreopsis*
*Roseicyclus marinus* Dej080120_10* (CP180348)	56	99.9	99.9	3,365,735	**3,365,935**	1	1	∼3.6	(Pseudomonadota) *Roseicyclus*
*Seohaeicola* sp. SP36* (CP180350)	40	99.7	99.7	4,282,476	**4,159,599**	5	1	∼4.5	(Pseudomonadota) EhC02 sp0016508895
*Thalassospira australica* NP 3b2 (JBLLNT000000000)	23	99.7	99.5	5,225,359	**4,831,494**	12	26	∼4.3	(Pseudomonadota) *Thalassospira*
*Thalassobaculum salexigens* A11D-306* (CP180353-4)	17	97.5	99.1	4,788,243	**4,768,590**	2	5	4.9 to 5.1	(Pseudomonadota) JANSYS01
*Polymorphobacter multimanifer* 262-7 (CP180346)	28	97.8	97.8	**2,817,605**	2,819,673	1	1	3.8 to 3.9	(Pseudomonadota) *Sandarakinorhabdus*
*Pikeienuella piscinae* RR4-56 (PRJNA1103200)	14	98.1	98.1	4,804,495	**3,776,009**	6	29	4.4 to 5.0	(Pseudomonadota) *Pikeienuella* sp030424705
*Yoonia vestfoldensis* SMR4r (CP180347)*^a^	6	99.4	NA	**3,172,507**	NA	1	NA	3.1 to 4.0	(Pseudomonadota) *Yoonia* sp018402175

Only coverage values for the more complete (bold) assembly are provided. Genome assemblies determined to be more complete are in bold. MAGs falling within the top 16 most abundant bacteria in the mixed host/parasite or naïve host culture are denoted by an *.

^a^The *Yoonia* MAG did not readily assemble from mixed host/parasite data, therefore the statistics provided are generated from naïve host data.

^b^Mean reads per base.

^c^Percent identity using BLASTn and the NCBI rRNA_typestrains/16S_ribosomal_RNA database.

^d^Typical genome size of MAG species or genus if unavailable.

**Table 3:** Bacterial MAGs assembling as single contigs from *Amoebophrya*-infected *K. veneficum* and uninfected *K. veneficum* cultures at lower abundance (not in top five in either culture) and without high-identity reference data

Contig label(s) (GenBank accession no.)	SILVA classification	Culture	ID (%)^a^	Top hit	Total length^b^	# Contigs	GC (%)	Coverage^c^	GTDB taxonomy
tig00000055 **(CP170360)**	Unclassified (Unknown E)	Infected	83.2	*Akkermansia muciniphila* ATCC BAA-835	7,152,164	1	63	54	(Verrucomicrobiota) GCA-2715965
tig00002473 **(CP170456)**	*Fluviicola*	Infected	94.9	*Fluviicola taffensis* DSM 16823	4,160,033	1	41	119	(Bacteroidota) UBA4466
tig00000052 **(CP170458)**	Unclassified (Unknown A)	Infected	93.7	*Marinoscillum furvescens* NBRC 15994	5,421,011	1	40	5	(Bacteroidota) UBA7330
tig00002477 **(CP170361)**	SM1A02 (A)	Infected	85.0	*Poriferisphaera corsica* KS4	3,148,344	1	65	13	(Planctomycetota) UBA1924
tig00000064 **(CP170363)**	OM182 clade	Infected	91.6	*Aestuariirhabdus litorea* GTF13	4,673,355	1	57	70	(Pseudomonadota) UBA9659 sp027621895
tig00000062 **(CP170364)**	Unclassified (Unknown F)	Infected	91.6	*Rhodocaloribacter litoris* ISCAR-4553	4,728,057	1	65	88	(Bacteroidota) JABDJZ01
tig00000070 **(CP170367)**	*Pseudohongiella*	Infected	94.5	*Pseudohongiella nitratireducens* SCS-49	3,997,272	1	54	59	(Pseudomonadota) UBA9145 sp027621405
tig00000072 **(CP170455)**	SM1A02 (B)	Uninfected	85.0	*Mucisphaera calidilacus* Pan265	3,411,796	1	61	110	(Planctomycetota) JANSXG01 sp024743075
tig00000056 **(CP170366)**	*Halioglobus*	Infected	93.7	*Chromatocurvus halotolerans* EG19	4,865,021	1	58	25	(Pseudomonadota) *Halioglobus* sp024742415
tig00000085 **(CP170362)**	Unclassified (Unknown D)	Uninfected	90.7	*Simkania negevensis* Z	2,603,172	1	45	26	(Chlamydiota) JACKPQ01

“Culture” refers to the culture in which the bacterium is more abundant.

^a^Percent identity using BLASTn and the NCBI rRNA_typestrains/16S_ribosomal_RNA database.

^b^Single contig length minus any overlap.

^c^Mean reads per base.

### Establishing a Core Microbial Community

**Fig. 2.**
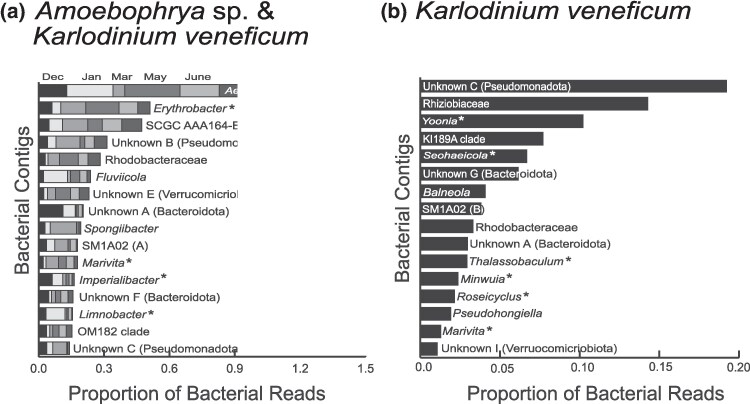
Relative abundance of the top 16 most abundant bacterial contigs in a) mixed host/parasite culture (81% of bacterial reads) across six sequencing runs (A to F) a month apart under the same culture conditions and in b) host-only culture (89% of bacterial reads) using just a single time point. Both estimates were obtained by mapping reads to a unified complete reference set of bacterial contigs. The complete or nearly complete MAGs based on comparisons with reference genome data are denoted by an asterisk (*).

There were 13 MAGs with at least 98% 16S rRNA gene sequence identity to cultured reference strains in the NCBI BLAST database (Table 2) and thus were representative of quite well-characterized bacterial species or genera. A cutoff of 98% identity of the 16S rRNA gene was selected as a compromise to the recent reassessment of the 97% identity clustering threshold and suggested value of 99% for full-length sequences (Edgar 2018). Four of these MAGs with good references fall within the community representing the top 16 most abundant bacteria in the mixed host/parasite culture, and six are abundant in the naïve host culture (Fig. 2a).

The 13 MAGs with good reference genomes were used to compare the results of the 2 assembly methods to an external reference. Between the two assemblers, Canu often generated the more complete genome in fewer contigs when compared with Flye, but overall, assembly success with either of the assembly algorithm was even, with 10 of the 13 selected MAGs assembling very similar regardless of algorithm employed. Although Flye yielded larger MAGs for *Thalassospira* and *Pikeienuella*, completeness and contamination values revealed these assemblies to contain more contamination than the corresponding Canu assemblies. Ultimately, four Flye-assembled genomes and nine Canu-assembled genomes were selected for functional analysis of MAGs with available reference genomes. Assembly and annotation statistics for these well-characterized (minimum of 98% 16S rRNA gene BLASTn identity) MAGs are provided in Table 2 and supplementary table S1, Supplementary Material online. Contamination values are minimal in every case except for the *Limnobacter* MAG (>15%), suggesting a possible overestimate of predicted roles in this genome.

The recent microbial survey of *Amphidinium carterae* was produced in the same lab, which could suggest potential contaminants, as well as a view into the shared bacterial genomes (Judd et al. 2024). High-identity (>97%) BLASTN matches were found for three MAGs. SM1A02 (B) from Table 3 was 99.9% identical (681 differences across 3.41 Mb) and contained 3 rearrangements outside of a 2.2-Mb contiguous match. This is likely a shared bacterial genome between the *A. carterae* and *K. veneficum* cultures that is relatively rare in both datasets. The second-best match was a MAG with 99.8% identity to *Seohaeicola* sp. that was less contiguous, including two 0.9 Mb matches, and the genome sizes slightly differ (4.27 Mb in the previous study versus 4.15 Mb in this study). This *Seohaeicola* sp. MAG is abundant in both datasets. The Rhizobiaceae that was relatively abundant in both cultures was also a good, if not exact, match (98.8% ANI) between the two strains, and the longest hits were 300 kb, suggesting more rearrangements between the two MAGs in this instance.

### Assessing Microbial Community Abundance in Mixed Host/Parasite and Naïve Host Cultures

In assessing the composition of the microbial community in the mixed host/parasite culture, read mapping indicates that 16 bacterial strains comprise 81% of the reads attributed to prokaryotes (Fig. 2a) and just 5 MAGs account for 47.2% of the data. The remaining 23 bacteria identified collectively account for 19% of the total bacterial reads sequenced and individually range in abundance from 0.2% (*Yoonia* sp.) to 2.2% (*Minwuia* sp.). Overall, the largest proportion of reads (21.2%) mapped to an *Aestuariibacter* sp. (Gammaproteobacteria), followed by an *Erythrobacter* sp. (Alphaproteobacteria) (8.5%), a bacterium of the order Pedosphaerales (SCGC AAA164-E04 sp.) (8.0%), an unclassified bacterium (HKST-UBA223 sp. according to GTDB) of the order Pseudomonadales (Gammaproteobacteria) (5.2%), and a Rhodobacteraceae (Alphaproteobacteria, ASV31 sp019061145 according to GTDB) (4.7%). A total of six rounds of culturing, DNA isolation, and sequencing were used on the mixed culture, allowing for comparison of read abundance along this time series. As an example, the dominant *Aestuariibacter* shows variable relative abundance and is relatively lower in in the March round of sequence data (5% of reads from that time point) and highest in the July round (43% of reads) but is consistently (five of six time points) a dominant and top ranked bacterial MAG. In the March dataset, four MAGs were more abundant than at any other time point but three were the second through fourth most abundant overall, while only one MAG (*Spongiibacter*), which was ranked ninth overall, had an abundance of 11% of the data at that one time point. This suggests overall stability with some stochastic elements. Similarly, the less abundant MAGs absent from Fig. 2 never appeared in any dataset at an abundance over 3%, reflecting another stable feature of the data. Conversely, some MAG had abundance values over 10% of the data in only a single time point including *Fluvicola*, Unknown A, and the *Spongiibacter* mentioned above. Overall, much of the community composition remains stable across these different sequence datasets. As this is the first study to investigate the bacterial community associated with *Amoebophrya* sp. ex *K. veneficum* and *K. veneficum*, there are no similar datasets against which to compare our findings. The taxonomic composition and stability of the dinoflagellate-associated bacteria may not translate to observations in the natural environment, due to a myriad of environmental factors, but is presented here as a necessary foundation for future studies.

In order to ascertain whether these bacteria associate with *K. veneficum* or the *Amoebophrya* parasite, bacterial contigs coassembling with *K. veneficum* grown in the absence of the parasite were also investigated. Interestingly, in the naïve host culture, the overall microbial community composition remains fairly consistent with all 39 MAGs recruiting at least some raw sequence reads in read mapping data from the host-only culture, but there is a clear rearrangement in abundance (Fig. 2b). In the *K. veneficum* culture absent of *Amoebophrya* parasite, the most abundant community member still belongs to the Gammaproteobacteria (19.2% of reads) but is classified in the Azotimanducaceae family. In the mixed host/parasite culture, this same bacterium comprises only 2% of the reads overall. The most abundant community member in the mixed culture, an *Aestuariibacter* sp., is ranked 31st in the host-only culture, and its abundance dropped dramatically from 21.2% to 0.2% of reads. In sum, 58.1% of the bacterial reads from the naïve host dataset assemble into the top five most abundant contigs. The top 16 most abundant bacteria in the uninfected *K. veneficum* culture shown in Fig. 2b account for 89% of the reads attributed to prokaryotes in this culture, and the remaining 23 bacteria identified individually range in abundance from <0.1% (Unknown H) to 1.1% (*Nioella* sp.). All the bacteria identified in the host-only culture are also present in the mixed host/parasite culture, further supporting the assertion that the bacterial community is associated with *K. veneficum*.

### Isolation of Cooccurring Bacteria

Attempts to culture bacteria identified from metagenomic sequencing resulted in the isolation of only two strains that were also identified in whole-culture sequencing (*Erythrobacter* sp. and *Limnobacter* sp.). *Erythrobacter* colonies were isolated from both *Amoebophrya*-infected and uninfected *K. veneficum* cultures, while *Limnobacter* was only isolated from the *Amoebophrya*-infected *K. veneficum* culture. The *Erythrobacter* sp. was the second most abundant bacterium in the mixed host/parasite culture as determined from read mapping. In contrast, the *Limnobacter* sp. comprises <3% of the total bacterial reads in either culture. Both strains were identified based on amplified 16S rRNA gene sequence analysis extracted from colonies on agar plates of Marine Agar 2216 and spent medium made from Chesapeake Bay water used for culturing *K. veneficum* combined with agar. Additionally, species belonging to the genera *Algoriphagus*, *Arenibacter*, *Maribacter*, *Marinobacter*, *Muricauda*, *Mycobacterium*, *Roseobacter*, and *Sphingopyxis* were recovered on Marine Agar 2216 from either mixed host/parasite or host-only cultures. Plating was performed with the specific intention of isolating bacterial species identified in the sequencing data and therefore was not robust. It is likely that more members of the cooccurring bacterial community are capable of isolation. Nevertheless, the repeated isolation of several distinct strains not identified during assembly underscores the commonly observed discordance between the microbial community profile as characterized from molecular analysis versus that characterized strictly from isolation experiments (Eckburg et al. 2005; Lagier et al. 2012; Rhoads et al. 2012; Montalvo et al. 2014; Wojno et al. 2020; Schoonbroodt et al. 2023).

The 16S rRNA gene of the *Erythrobacter* sp. MAG is 100% identical to that of the cultured *Erythrobacter* isolate and the 1,492-bp 16S rRNA gene sequences share 98.9% identity with *Erythrobacter aurantius* strain *C5* in NCBI. The 16S rRNA gene of the *Limnobacter* isolate is 100% and 99.85% identical to the two copies present in the assembled MAG, and the 1,377-bp 16S rRNA gene sequence shares 100% identity with *Limnobacter alexandrii* strain LZ-4 in NCBI. To verify strain identity, we selected one of the isolates (*Erythrobacter* sp.) for additional analysis with whole -genome sequencing. Interestingly, visual comparison of the two genomes reveals a significant portion to be inverted (supplementary fig. S1, Supplementary Material online). This rearrangement is likely due to imperfect MAG assembly and the slight contamination (1.3%) remaining in the assembly, or genuine diversity in genome arrangement within the *Erythrobacter* population. The *Erythrobacter* sp. MAG assembly is slightly larger (3.36 Mb) than the corresponding isolates (3.27 Mb). Despite this discrepancy, identity is supported by whole-genome ANI values, calculated by both BLAST+ (ANIb of isolate to MAG = 100 or 99.60% of nucleotides) and MUMmer (ANIm of isolate to MAG = 99.99 or 99.86% of nucleotides). Therefore, we conclude that the *Erythrobacter* sp. identified from sequencing data is in fact identical to the cultivated strain. This result also confirms the relatively low error rate on a whole-genome scale of the sequencing and assembly approach.

### Functional Analysis of MAGs and Most Abundant Contigs

**Fig. 3.**
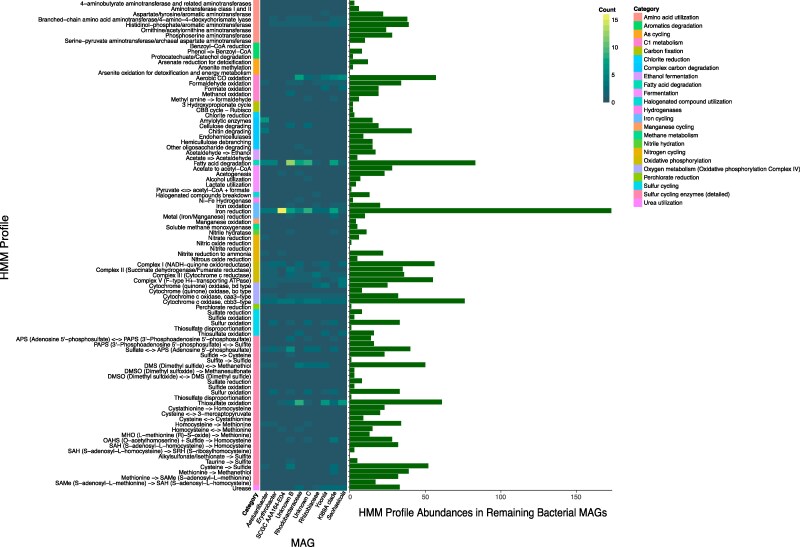
HMM profiles detected individually in the most abundant bacterial MAGs and collectively in the remaining 29 bacterial MAGs.

**Fig. 4.**
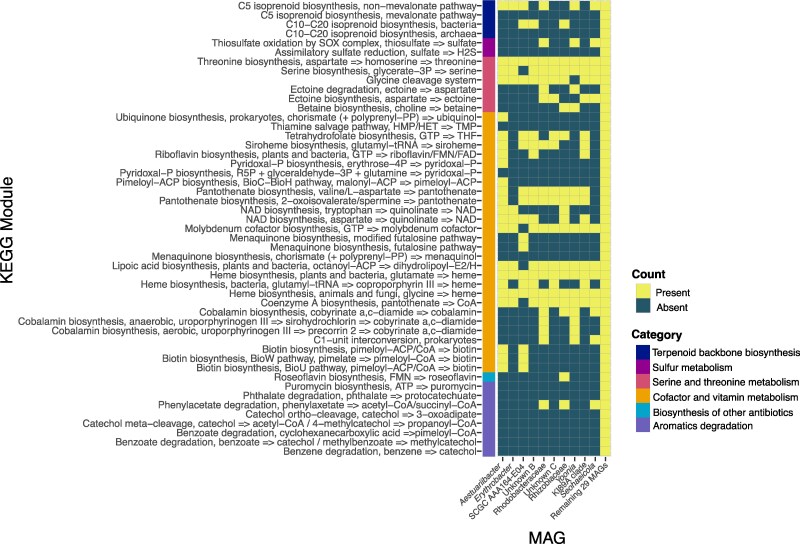
KEGG modules detected individually in the most abundant bacterial MAGs and collectively in the remaining 29 bacterial MAGs.

**Fig. 5.**
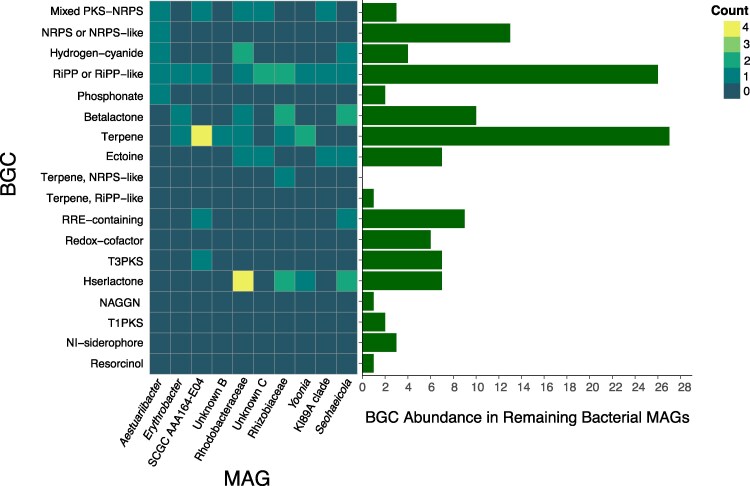
BGCs detected individually in most abundant bacterial MAGs and collectively in the remaining 29 bacterial MAGs. Generated by antiSMASH. Note: Two BGCs are double represented by the “riPP recognition element (RRE)-containing” category.

The MAG data were then mined for hidden markov model (HMM) profiles, kyoto encyclopedia of genes and genomes (KEGG) orthologs, and Biosynthetic Gene Clusters (BGCs) as annotated by antiSMASH. Overall, there is a general correlation between the HMM profiles of the most abundant (top five in either culture) MAGs and lesser abundant MAGs (Fig. 3). The HMM profiling reveals that many of the bacterial strains retain pathways were associated with sulfur metabolism, iron reduction, oxygen metabolism, and fatty acid degradation. Iron reduction is the most frequently annotated pathway, followed collectively by various pathways associated with metabolism of organic sulfur. Annotation of KEGG orthologues supports a variety of abilities within the bacterial community to metabolize amino acids, vitamins, cofactors, and terpenoids (Fig. 4). In all cases where a pathway is absent in a particular MAG or contig, the pathway is present in the collection of contigs pertaining to less abundant species summarized as “remaining bacterial contigs” in the last column in Fig. 4. The prevalence of pathways for terpene synthesis is supported by BGC analysis (Fig. 5).

## Discussion

Here, we present a deep and comprehensive survey of the bacteria associated with *Amoebophrya* sp. ex *K. veneficum* and a smaller dataset of bacteria associated with *K. veneficum*. The goal of this survey is to provide detailed, complete genomes for bacteria from these cultures that can complement and reinforce data from amplicon surveys and environmental sequencing. As there are no other studies documenting bacterial associations with this parasite or the host, the significance of these associations can only be inferred from the roles that the bacteria identified here play in relationships with other plankton hosts, which range from stochastic to mutualistic or parasitic (Menzel and Spaeth 1962; Silva and Franca 1985; Silva 1990; Fukami et al. 1992; Doucette 1998; Elbrächter and Schnepf 1998; Yoshinaga et al. 1998; Kirchner et al. 1999; Coats and Park 2002). In dinoflagellates, symbiotic bacteria may provide essential nutrients (phosphorus [P] and nitrogen [N]), vitamins, and hormones supporting growth of the host (Menzel and Spaeth 1962; Haines and Guillard 1974; Swift and Guillard 1978; Axler et al. 1981; Maruyama et al. 1986; Doucette 1995; Croft et al. 2005; Wang et al. 2017). Bacteria have also been reported to stimulate dinoflagellate toxin production; however, the mechanism by which this effect is carried out remains elusive (Gallacher et al. 1997; Hold et al. 2001; Uribe and Espejo 2003; Green et al. 2010). Certain bacteria may autonomously synthesize these toxins, although lack of consensus in subsequent studies casts doubt on this possibility (Kodama et al. 1988; Orr et al. 2013). The ability of some bacteria to degrade host-produced toxins (Bai et al. 2007; Deng et al. 2023) and secrete algicidal compounds (Fukami et al. 1991; Imai et al. 1993; Doucette 1995; Lovejoy Connie et al. 1998; Lu et al. 2016; Cho et al. 2021) or growth-inhibiting metabolites (Ishio 1989; Sawayama et al. 1991, 1993; Onishi et al. 2021) may modulate host toxicity and support *Amoebophrya* sp. ex *K. veneficum* infection. Finally, the bacteria in this culture may simply represent stochastic or opportunistic interactions as the dinoflagellate hosts are lysed *en masse* during parasite emergence.

From our analysis, 39 unique bacteria were identified in the *Amoebophrya-*infected *K. veneficum* and uninfected *K. veneficum* cultures, and 28 of these members comprise the majority of the community (Fig. 2) in the two cultures. From the core community of 39 MAGs, we can definitively identify 13 as well-characterized, of which ten genera (*Imperialibacter*, *Marivita*, *Roseicyclus*, *Seohaeicola*, *Erythrobacter*, *Nioella*, *Thalassobaculum*, *Limnobacter*, *Thalassospira*, and *Yoonia*) have previously been documented with marine algae, including dinoflagellates (Lu et al. 2016; Wang et al. 2017; Deschaseaux et al. 2019; Johansson et al. 2019; Duan et al. 2020; Karimi et al. 2020; Yang et al. 2020; Zhou et al. 2021; Deng et al. 2023; Martínez-Mercado et al. 2024). Eight of the ten bacterial strains identified from culturing efforts (*Algoriphagus*, *Arenibacter*, *Erythrobacter*, *Limnobacter*, *Maribacter*, *Marinobacter*, *Muricauda*, and *Roseobacter*) have also been associated with dinoflagellates (Alavi et al. 2001; Hold et al. 2001; Bowman et al. 2003; Green et al. 2004; Miller and Belas 2004; Nedashkovskaya et al. 2004a, 2004b; Green et al. 2006; Kodama et al. 2006; Alegado et al. 2011; Wang et al. 2017; Lawson et al. 2018; Chen et al. 2019; Hattenrath-Lehmann et al. 2019; Duan et al. 2020; Fu et al. 2020; Chorazyczewski et al. 2021; Maire et al. 2021; Takagi et al. 2023). While some of these bacteria, such as *Yoonia* spp. and *Marinobacter* spp., have been observed to stimulate growth of their respective hosts (Johansson et al. 2019; Yang et al. 2021), others, including *Thalassospira* spp., has been shown to exhibit algicidal activity (Lu et al. 2016; Chen et al. 2020; Deng et al. 2023).

Bacteria have previously been cited for roles in maintaining algal fitness and survival based on genome evidence of pathways for production of vitamins (Karimi et al. 2020), carotenoids (Motone et al. 2020; Takagi et al. 2023), siderophores (Amin et al. 2009; Takagi et al. 2023), superoxide radical degradation, amino acid transport/metabolism, and carbohydrate metabolism (Zhou et al. 2021) in these microbes. Metabolic potential related to amino acids and carbohydrates suggests cross-kingdom interactions through nutrient exchange or signaling that may facilitate improved host fitness, or, conversely, indicate bacterial exploitation of host resources to the detriment of the host (Cole 1982; Seymour et al. 2017). Additionally, evidence of microbially mediated phosphorus utilization demonstrates selection for bacterial species with improved ability to exploit P sources within a dinoflagellate-associated microbial community (Wang et al. 2017). Toxicity of *K. veneficum* populations varies over time and intensifies under growth-limiting conditions due to low N and P (Adolf et al. 2009). Therefore, it is possible that by facilitating dinoflagellate growth, these bacteria may also contribute to lower karlotoxin production. Notably from the group of isolates cultured from the *Amoebophrya-*infected *K. veneficum* and uninfected *K. veneficum* cultures, *Roseobacter* maintain a strong surface association with dinoflagellates due to their ability to rapidly degrade dimethylsulfoniopropionate (DMSP), of which dinoflagellates are a major producer (Miller and Belas 2004). In addition, sequence similarity between dinoflagellate minicircles (components of dinoflagellate plastid genomes) and *Algoriphagus* spp. provides strong support for horizontal gene transfer and indicates a close association between some bacteria and dinoflagellate hosts (Moszczynski et al. 2012). In considering the protective scavenging effects that pigments can impart on their producer/host, it should be noted that all of the bacteria isolated from the mixed host/parasite and host-only cultures, aside from *Roseobacter* (white) and *Marinobacter* (iridescent), are pigmented orange, pink, or yellow.

From the metabolic pathways identified in the most abundant MAGs, we can speculate on the significance of their associations with *K. veneficum* and/or *Amoebophrya* sp. ex *K. veneficum.* HMM profiling reveals many of the bacterial strains retain pathways associated with sulfur metabolism, iron reduction, oxygen metabolism (specifically cytochrome c oxidase), and fatty acid degradation. Iron reduction is the most frequently annotated pathway, followed by pathways associated with organic sulfur metabolism. Bioavailability of iron affects dinoflagellate growth, and pulsed inputs of iron may trigger HABs (Wells et al. 1991; Mitrovic et al. 2004). Surprisingly, while it is believed that microbially produced siderophores supply iron to the dinoflagellate (Yarimizu et al. 2018), only three siderophore BGCs were annotated in the entire bacterial dataset, all belonging to lesser abundant and fragmented MAGs. The discrepancy between iron reduction capabilities and siderophore production may suggest an insignificant role of the cooccurring bacteria in providing iron for *K. veneficum* in culture. The prevalence of genes related to sulfur metabolism and dimethyl sulfoxide (DMSO) metabolism reflect the sulfur-rich environment associated with HABs and the ability of these microbes to degrade DMSP. As a major producers of DMSP in the marine environment, dinoflagellates contribute significantly to the global biosulfur cycle (Yoch 2002; Lin et al. 2021), and studies show that DMSP levels can alter the composition of the associated bacterial community (Lin et al. 2021). Bacteria with a higher proportion of cytochrome c may be better suited to coexisting with toxic dinoflagellate blooms, since this protein also functions as an antioxidant and can thus neutralize reactive oxygen species (ROS) generated during HABs (Hattenrath-Lehmann et al. 2019). Whether bacteria with this capability are selected for by the dinoflagellate host, or if this is an adaptation to their environment, is unknown. Future studies should investigate whether the bacterial community described in this study changes during a *K. veneficum* bloom, when concentrations of DMSP and ROS rise. These data would further aid in assessing the stability of this community and the degree of mutualism between *K. veneficum* and cooccurring bacteria.

Looking at the KEGG modules of interest, it is apparent that many of the well-assembled MAGs and most abundant contigs assembled from the dinoflagellate cultures retain pathways associated with metabolism of amino acids (serine and threonine), cofactors and vitamins, isoprenoid biosynthesis, as well as the degradation of aromatics. Vitamins B1 (also known as thiamine), B7 (also known as biotin), and B12 (also known as cobalamin) are essential for carbon metabolism, fatty acid metabolism, and amino acid metabolism, respectively, but many dinoflagellates are incapable of synthesizing these cofactors (Cruz-López and Maske 2016). These vitamins, including vitamin B2 (also known as riboflavin), also function as antioxidants, and have been shown to increase in *K. veneficum* under oxidative stress (Wang and Coyne 2022). Therefore, bacteria capable of synthesizing these vitamins may provide a critical service to their dinoflagellate host. Interestingly, a minority (zero to three spp. in each case) of the most abundant contigs identified are capable of synthesizing these vitamins, highlighting the significant contributions of the less abundant community members and underscoring a need to characterize these elusive microbes. This need is further emphasized by the fact that in several cases, pathways pertaining to the degradation of specific aromatics, terpenoid biosynthesis, sulfur metabolism, and cofactor metabolism are lacking in all of the most abundant MAGs, but present in less abundant bacterial contigs that were identified during assembly. Tetrahydrofolate biosynthesis capabilities in the majority of the most abundant bacterial contigs demonstrate the potential to take advantage of the abundant DMSP that is released by dinoflagellates during blooms (Miller and Belas 2004). A complete absence of glycan biosynthesis pathways in any of the assembled bacterial contigs assembled suggests that these bacteria are not likely endosymbionts, as glycan–lectin interactions are typically essential for the recognition and uptake of symbionts (Tivey et al. 2020; Matthews et al. 2023). Of the most abundant community members, *Yoonia*, is the sole MAG capable of degrading the osmolyte ectoine, which may be antagonistic to *K. veneficum* survival under stress, although further research is required to verify this hypothesis. Ultimately, the presence/absence nature of this analysis precludes studies of relative abundance among bacterial community members. It remains unknown whether the partial presence of certain pathway components (for biotin, tetrahydrofolate, as well as a module associated with sulfate reduction) indicates incomplete pathways or the presence of multiple alternative biosynthetic pathways for various metabolites. Future studies investigating the activity of coassembling bacteria from dinoflagellate cultures should link metabolomic and transcriptomic data to genomic analysis to verify the production of these metabolites.

Analysis of BGCs present in the bacteria coassembling with *K. veneficum* and the *Amoebophrya* parasite reveals terpenes, ribosomally synthesized and posttranslationally modified peptides, betalactones, and hserlactones to be the most commonly annotated BGCs in this dinoflagellate–parasite–microbe complex. The high proportion of terpenes is likely associated with the production of antioxidants that protect the bacteria from ROS produced by the dinoflagellate host(s). These secondary metabolites may play a role in chemical defense of the dinoflagellate host against predators, as well as intermicrobial and host–microbe communication. Metabolic analysis of coassembling bacteria from the brown algae *Ectocarpus subulatus* reveals similar patterns of functional annotation (Karimi et al. 2020).

Consistent reports of bacterial genera cooccurring with dinoflagellates contribute to a deeper understanding of a putatively core microbial community and highlight the need to investigate host–microbe dynamics for their possible role in mediating HABs. The community identified in this study stems from a single cultured *K. veneficum* bloom isolated from the Chesapeake Bay many years ago, and to confirm that the microbial community identified from this cultured system translates to observations in the wild, analysis of the natural bacterial composition at *K. veneficum* collection sites will be required. Nevertheless, the identification of strains such as *Marivita* sp. CB1101, which was originally isolated from the Chesapeake Bay (Budinoff et al. 2011), and the culturing of *Marinobacter*, which has been reported from geographically distant laboratory cultures of other dinoflagellates (Green et al. 2006), strengthen the reality of these findings. Furthermore, the small overlap between dominant members of the bacterial community described here and of those associated with other dinoflagellates cultured in the same laboratory is evident against contamination (Judd et al. 2024). Despite the fact that the dominating bacterial strains in these cultures must have competitive advantages over other community members, we cannot assign a genus to the majority of the most abundant MAGs. Characterization of these novel strains is essential to understanding the significance of their associations with dinoflagellates. It is critical to keep in mind that metagenome assembly is not foolproof and carries several biases. Accuracy of assembly and binning techniques diminishes for repetitive regions of the genome and for Guanine and Cytosine (GC) or Adenine and Thymine (AT) base rich sequences (Nelson et al. 2020). Mobile genetic elements may confound assemblers and consequently be discarded or incorrectly binned. The presence of closely related species within a community may be obscured by the compression of highly conserved regions such as ribosomal RNA operons by assembly or binning algorithms (Nelson et al. 2020), as is likely the case with *Thalassospira* in this analysis and additionally demonstrated by the rearrangements between the cultured and MAG *Erythrobacter* genomes. Conversely, bacteria present in low abundances may also go undetected if the corresponding contigs sequenced lack 16S rRNA genes. Furthermore, it is more difficult to assess the accuracy of a MAG without a strong reference genome. To minimize biases with the approach to characterizing the bacterial community exclusively from Nanopore data, thorough cross-validation of results was performed using two assemblers (Flye and Canu), two taxonomic classifiers (SILVA and GTDB), and three quality metrics (EvalG of Bacterial and Viral Bioinformatics Resource Center [BV-BRC], ANI, and CheckM2). Additional studies on *K. veneficum*-associated bacterial communities and repeated discovery of similar MAGs are necessary to verify the “biological reality” of the elusive MAGs identified in this study (Nelson et al. 2020; Setubal 2021). The importance of cultivation-based strategies also must not be overlooked in continuing community characterization studies, as evident by the nearly disparate communities identified between metagenomic data and cultivation studies seen here. While there will always remain a degree of uncertainty around MAG identification, comprehensive analysis of metabolic capabilities of the bacterial community is less ambiguous and provides critical insight into the dynamics between bacteria and dinoflagellate host(s).

It remains to be explored how these bacteria establish their associations with *K. veneficum* or whether they in fact cooccur with *Amoebophrya* sp. ex *K. veneficum*. Nevertheless, consistencies in the microbial community between the mixed host/parasite culture and naïve host culture strongly suggest that these bacteria associate more with *K. veneficum* than the *Amoebophrya* parasite. Future studies should investigate the relationship between the rearrangement in bacterial community structure and metabolic capabilities observed in infected versus noninfected *K. veneficum* cultures over the cycle of infection to better clarify the significance of these associations. Coats and Park (2002) posited that bacteria could serve as a nutrition source for *Amoebophrya* sp. ex *K. veneficum* dinospores, thus prolonging survival of the parasite outside of a host, although this was never tested (Park et al. 2013). The inability to study *Amoebophrya* sp. ex *K. veneficum* in isolation precludes any definitive conclusions as to which dinoflagellate these persistent bacteria associate with, but it is certain that their presence is mediated by multivariable and complex processes, including cross-kingdom interactions (host–microbe and parasite–microbe), intermicrobial competition, and stochastic factors.

## Materials and Methods

### Dinoflagellate/Parasite Cultivation

Host cultures of *K. veneficum* were grown in f/2 -Si media made from local seawater diluted to a salinity of 15 at 20 °C with 100 microEinsteins of light with 14 h light and 10 h dark. Parasite cultures with 100,000 dinospores per mL were combined with host cultures at 10,000 cells per mL and diluted with fresh media to 50,000 dinospores per mL and 5,000 host cells per mL and then grown for 7 d. A Coulter counter was used to count and distinguish dinospores (2 to 4 μm) and host (10 to 13 μm) prior to changing the cultures. Each week, the cultures were changed and DNA was isolated from the excess volume.

### *Amoebophrya* Sequencing Methods

Week-old cultures that were dominated by dinospores based on Coulter counts were centrifuged at 9,000 × *g* for 10 min and then lysed with a 2% cetyltrimethylammonium bromide (CTAB), 1.4 M NaCl, 10 mM ethylenediaminetetraacetic acid, and 100 mM Tris pH 8.0 solution preheated to 50 °C with 25 U/mL of proteinase K at 50 °C for 20 min with gentle agitation. The lysate was extracted twice with equal volumes of chloroform with centrifugation at 10,000 × *g* for 10 min to separate the aqueous phase. The aqueous phase was then mixed with Zymocat binding buffer (Zymo Research, Irvine, CA, USA) and purified according to the manufacturer's protocol. The Short Read Eliminator kit (Pacific Biosciences, Menlo Park, CA, USA) was used to isolate higher molecular weight DNA with Nanodrop (Thermo Fisher Scientific, Waltham, MA, USA) and Qubit (Thermo Fisher Scientific) methods used to quantify the DNA. The DNA was then sequenced using the Ligation Sequencing Kit V14 protocol (Oxford Nanopore Technologies, Oxford, UK) with DNA repair using the NEB Companion Sequencing Module (New England Biolabs, Ipswich, MA, USA). Sequencing was performed on the version 10 chip using a MinION instrument, and libraries were reloaded up to three times on a single sequencing chip to obtain maximum sequence yield. Sequences were base called using Dorado with the “super” accuracy model (Model: dna_r10.4.1_e8.2_400bps_sup@v4.2.0). With the goal of obtaining more sequence data, culturing, DNA isolation, and sequencing were performed six times over a span of 7 months representing the months as shown in Fig. 2.

### MAG Assembly and Binning

Reads were assembled using both Flye version 2.9.1-b1780 (Lin et al. 2016; Kolmogorov et al. 2019) and Canu version 2.2 (Koren et al. 2017), and the more complete assembly was used for further analysis. Initially, contigs were manually binned into MAGs based on identical 16S rRNA gene sequence alignment results, as well as alignment of Canu to Flye assembly. Next, the MaxBin2 tool (Wu et al. 2014) was used to bin genomes based on GC content and coverage.

### Taxonomic Characterization and Functional Analysis

The quality of each bin was assessed using the GTDB-Tk v2 tool with release 220 of the database (Chaumeil et al. 2022). The bins from each assembly method were identified based on 16S rDNA, so that bins from the Flye and Canu assemblers could be compared. Flye and Canu bins were compared with each other using BLASTN (BLASTN, RRID:SCR_001598) at >97% sequence identity to remove spurious contigs and the control DNA used in Nanopore library prep as well as to select the bin with fewer contigs between the two assemblers used. Each bin was then checked again using GTDB-Tk v2 and CheckM2 (Chklovski et al. 2023) to confirm that completeness and contamination values were improved or identical. Genome completeness was also evaluated by comparison with reference strains in the literature to assess which MAGs likely represent complete or near-complete genome assemblies. MAGs were annotated using the BV-BRC (Olson et al. 2023). Functional analyses of bacterial contigs as annotated by hmmsearch and KOfam were performed using METABOLIC-G version 4.0 with default settings (Zhou et al. 2022). BGCs were identified using antiSMASH 7.0 (Blin et al. 2023).

### Estimating Abundance of MAG Contigs

Abundance estimates were performed using a complete, unified reference set of the 39 bacterial MAGs composed of 294 sequences including the 3 sequences assembled from the naïve host data and the remainder was assembled from infected cultures. Reads from the naïve host or the six different mixed host/parasite rounds of DNA sequencing were mapped to the reference set using minimap2 (Li, 2018) excluding secondary mapping. After sorting and removing supplemental mapped reads and reads with a mapping quality <50, the samtools coverage command (Danecek et al. 2021) was then used to generate read count information for abundance measurements. The relative abundance was calculated as the number of reads for each contig divided by the total number of bacterial reads in that dataset.

### Phylogenetic Analysis of Bacterial MAGs

After assembly, putative bacterial contigs were detected using BLASTN against the rRNA_typestrains/16S_ribosomal_RNA database from NCBI. These candidate bacterial contigs were screened to remove lower identity organellar and eukaryotic ribosomal sequences and then annotated. Taxonomic assignment was also performed by using the GTDB-Tk classify_wf and GTDB-Tk de_novo_wf methods based on protein-coding genes or the ANI method. Comparisons between bacterial contigs assembled with the two different assemblers were done using the D-Genies web application (D-GENIES, RRID:SCR_018967) (Cabanettes and Klopp 2018) and BLASTN.

### Isolation of Cooccurring Bacteria

Aliquots of *Amoebophrya*-infected *K. veneficum* and uninfected *K. veneficum* cultures were serially diluted in sterile MilliQ water, and 100 μL of each dilution was plated on Marine Agar 2216 and sterilized spent cultivation media (f/2 -Si at a salinity of 15) with 20% agar, in triplicate. Plates were incubated at 30 °C until visible colonies were formed. Unique colonies were restreaked onto Marine Agar 2216 (Burlington, MA, USA) until confirmed to be pure. From these plates, two individual colonies were selected for DNA extraction using the DNeasy UltraClean Microbial DNA Isolation Kit (Qiagen, Germantown, MD, USA). Partial 16S rRNA gene fragments were amplified using degenerate primers 27F (Hyman et al. 2005) and 1492R (Sasoh et al. 2006) and sequenced by the Sanger method. Isolates were identified by comparing partial 16S rRNA gene fragments against the NCBI database with BLASTN.

### Whole-Genome Sequencing and Assembly of *Erythrobacter* Isolate

Two colonies from a pure plate of the *Erythrobacter* isolate were transferred to 10 mL Marine Broth 2216 and incubated at 30 °C with shaking at 140 RPM until dense growth was visible. DNA was extracted using the PacBio Nanobind CBB High Molecular Weight for Gram Negative protocol, and DNA yield was quantified using Qubit. A sequencing library was prepared with the Native Barcoding Kit 24 V14 (SQK-NBD114.24, Oxford Nanopore Technologies) and sequenced on an R10.4.1 flow cell on a MinION device at 400 bases per second. Base calling was performed with Dorado (model dna_r10.4.1_e8.2_400bps_sup@v4.2.0), and reads were demultiplexed with Porechop version 0.2.4 (https://github.com/rrwick/Porechop). Read quality was assessed with FastQC version 0.12.0 (Andrews 2010), and 490,392 reads were originally generated with 2.498 billion bases. A size-filtered subset of the raw reads (≥18 kb) was used for assembly with Flye version 2.9.1-b1780 to reduce coverage and generate one contig. Circlator version 1.5.5 was used to circularize the genome and to set the origin of replication (Hunt et al. 2015).

Pairwise genome comparison of the *Erythrobacter* with to the *Erythrobacter* MAG was performed by calculating ANI based on BLAST and MUMmer (MUMmer, RRID:SCR_018171) with JSpecies Web Server (JSpeciesWS) (JSpeciesWS, RRID:SCR_022059) (Kurtz et al. 2004; Goris et al. 2007; Richter et al. 2016). D-Genies web application (D-GENIES, RRID:SCR_018967) was used to create a dot plot, using minimap2 version 2.26 (minimap2, RRID:SCR_018550) (Cabanettes and Klopp 2018; Li, 2018) for alignment.

## Supplementary Material

evaf022_Supplementary_Data

## Data Availability

All the bacterial contigs have been submitted to GenBank under BioProject accession no. PRJNA1103200 and SRA accession nos. SRR28830816-18 and SRR28782276-78.
