# Supplementary material for: Nanopore Sequencing of Amoebophrya Species Reveals Novel Collection of Bacteria Putatively Associated With Karlodinium veneficum
Source: Genome Biol Evol. 2025 Feb 13;17(3):evaf022. doi: 10.1093/gbe/evaf022 (PMC11890096; doi:10.1093/gbe/evaf022)
Supplement: evaf022_Supplementary_Data [file evaf022_supplementary_data.zip › Tizabi_GBE_Supplementary_Table_S1_12302024.docx]

**Supplementary Table S1.** Annotation details of the best characterized (minimum of 98% 16S rRNA gene BLASTn identity) MAGs^a^ from *Amoebophrya*-infected *K. veneficum* culture.

| **MAG BLAST ID**  **(GenBank Accession No.)** | **Assembly Method** | **Completeness** | **Contamination** | **GC** | **CDS** | **tRNA** | **rRNA** | **GTDB Taxonomy** |
| --- | --- | --- | --- | --- | --- | --- | --- | --- |
| *Erythrobacter aurantius* strain C5* | Canu | 99.6% | 1.3% | 62% | 3,277 | 45 | 3 | (Pseudomonadota) *Erythrobacter aurantius* |
| *Marivita* sp. CB1101* | Canu | 100% | 0% | 60% | 4,452 | 47 | 9 | (Pseudomonadota) *Marivita* sp030148515 |
| *Imperialibacter* sp. Rs* | Canu | 95.9% | 4.1% | 47% | 6,104 | 41 | 4 | (Bacteroidota) *Imperialibacter* |
| *Limnobacter thiooxidans* CS-K2 | Flye | 91.3% | 15.6% | 52% | 3,470 | 38 | 6 | (Pseudomonadota) *Limnobacter* sp902506325 |
| *Minwuia thermotolerans* SY3-13* | Flye | 100% | 0% | 66% | 4,450 | 49 | 6 | (Pseudomonadota) *Minwuia* |
| *Nioella ostreopsis* Z7-4 | Canu | 100% | 0% | 63% | 4,962 | 49 | 9 | (Pseudomonadota) *Nioella ostreopsis* |
| *Roseicyclus marinus* Dej080120_10* | Canu | 100% | 2% | 66% | 3,349 | 42 | 3 | (Pseudomonadota) *Roseicyclus* |
| *Seohaeicola* sp. SP36* | Canu | 100% | 0% | 64% | 4,179 | 63 | 6 | (Pseudomonadota) EhC02 sp0016508895 |
| *Thalassospira australica* NP 3b2 | Canu | 97.7% | 2.9% | 54% | 4,680 | 65 | 15 | (Pseudomonadota) *Thalassospira* |
| *Thalassobaculum salexigens* A11D-306* | Canu | 99.3% | 3.9% | 67% | 4,690 | 49 | 6 | (Pseudomonadota) JANSYS01 |
| *Polymorphobacter multimanifer* 262-7 | Flye | 100% | 0% | 67% | 2,707 | 45 | 3 | (Pseudomonadota) *Sandarakinorhabdus* |
| *Pikeienuella piscinae* RR4-56 | Canu | 100% | 0% | 69% | 3,840 | 38 | 3 | (Pseudomonadota) *Pikeienuella* sp030424705 |
| *Yoonia vestfoldensis* SMR4r*^b^ | Flye | 99.1% | 3.5% | 60% | 3,316 | 45 | 6 | (Pseudomonadota) *Yoonia* sp018402175 |

Note: MAGs falling within the top 16 most abundant bacteria in the mixed host/parasite or naïve host culture are denoted by an asterisk (*). ^a^ Statistics are provided for the more complete assembly for each MAG (Flye v. Canu, as determined in Table 2 in bold); ^b^ The *Yoonia* MAG did not readily assemble from mixed host/parasite data, therefore the statistics provided are generated from naïve host data.
