# Supplementary material for: Nanopore Sequencing of Amoebophrya Species Reveals Novel Collection of Bacteria Putatively Associated With Karlodinium veneficum
Source: Genome Biol Evol. 2025 Feb 13;17(3):evaf022. doi: 10.1093/gbe/evaf022 (PMC11890096; doi:10.1093/gbe/evaf022)
Supplement: evaf022_Supplementary_Data [file evaf022_supplementary_data.zip › Tizabi_GBE_Supplementary_Table_S2_12302024.docx]

**Supplementary Table S2.** Bacterial MAGs isolated from *Amoebophrya*-infected *K. veneficum* and uninfected *K. veneficum* cultures requiring extensive binning (> 1 contig) and of low abundance.

| **Contig Label(s)**  **(GenBank Accession No.)** | **SILVA Classification** | **Culture** | **ID**^a^ | **Top Hit** | **Total Length**^b^ | **# Contigs** | **Completeness**^c^ | **Contamination^c^** | **GC** | **Coverage**^d^ | **GTDB Taxonomy** |
| --- | --- | --- | --- | --- | --- | --- | --- | --- | --- | --- | --- |
| multiple  **()** | C1-B045 | uninfected | 92.6% | *Spongiibacter nanhainus* CSC3.9 | 3,862,041 | 8 | 95.83% | 11.87% | 51% | 16 | (Pseudomonadota) GM7GCV1 |
| multiple  **()** | Unclassified (Unknown G) | infected | 86.7% | *Pseudopedobacter saltans* DSM 12145 | 4,025,918 | 8 | 98.6% | 4.92% | 36% | 25 | (Bacteroidota)  SB11 |
| multiple  **()** | Unclassified (Unknown H) | infected | 90.4% | *Halobacteriovorax vibrionivorans* BL9 | 3,086,932 | 7 | 100% | 0.43% | 42% | 24 | (Bdellovibrionata) UBA4207 |
| multiple  **()** | *Peredibacter* | infected | 91.7% | *Halobacteriovorax marinus* SJ | 2,967,923 | 33 | 76.92% | 1.37% | 42% | 23 | (Bdellovibrionata) UBA4207 |
| multiple  **()** | *Balneola* | uninfected | 94.2% | *Balneola vulgaris* strain 13IX/A01/164 | 3,177,734 | 29 | 90.33% | 0.12% | 40% | 18 | (Bacteroidota) RHLJ01 |
| multiple  **()** | *Oleiphilus* | infected | 93.3% | *Amphritea pacifica* ZJ14W | 2,834,209 | 26 | 73.91% | 0.97% | 44% | 18 | (Pseudomonadota) PZPK01 |
| multiple  **()** | Unclassified (Unknown I) | infected | 91.2% | *Lacunisphaera limnophila* IG16b | 4,160,573 | 6 | 83.47% | 1.66% | 59% | 14 | (Verrucomicrobiota) *Synoicihabitans* |
| multiple  **()** | Sva0996 marine group | infected | 91.2% | *Rhabdothermincola sediminis* SYSU G02662 | 3,568,716 | 23 | 87.37% | 1.55% | 69% | 14 | (Actinomycetota)  JAJRXC01 |
| multiple  **()** | *Methylotenera* | infected | 95.6% | *Methylotenera oryzisoli* strain La3113 | 2,191,283 | 33 | 81.76% | 4.35% | 41% | 15 | (Pseudomonadota) GCA-2401735 |

Note: “Culture” refers to the culture in which the bacterium is more abundant. ^a^ % identity using BLASTn and the NCBI rRNA_typestrains/16S_ribosomal_RNA database; ^b^ Single contig length minus any overlap; ^c^ CheckM2 values; ^d^ Mean reads per base.
