# Supplementary figures and images for: Nanopore Sequencing of Amoebophrya Species Reveals Novel Collection of Bacteria Putatively Associated With Karlodinium veneficum
Source: Genome Biol Evol. 2025 Feb 13;17(3):evaf022. doi: 10.1093/gbe/evaf022 (PMC11890096; doi:10.1093/gbe/evaf022)

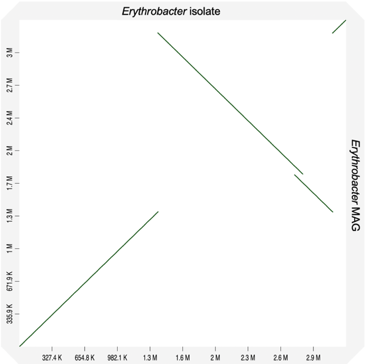


**Supplementary Figure S1.** Dot plot of *Erythrobacter* MAG v. cultured *Erythrobacter* isolate.

Supplement: evaf022_Supplementary_Data [file evaf022_supplementary_data.zip › Tizabi_GBE_Supplementary_Figure_S1_12302024.docx]
